# *catena*-Poly[[trimethyltin(IV)]-μ-2-methylbenzoato-κ^2^ *O*:*O*′]

**DOI:** 10.1107/S1600536809051587

**Published:** 2009-12-04

**Authors:** Muhammad Danish, Iram Saleem, Nazir Ahmad, Wojciech Starosta, Janusz Leciejewicz

**Affiliations:** a Department of Chemistry, University of Sargodha, Sargodha 40100, Pakistan; b Institute of Nuclear Chemistry and Technology, ul. Dorodna 16, 03-195 Warszawa, Poland

## Abstract

The polymeric structure of the title compound, [Sn(CH_3_)_3_(C_8_H_7_O_2_)]_*n*_, is composed of zigzag chains in which the tin(IV) atoms, coordinated by three methyl groups, are bridged by toluene-2-carboxyl­ate ligands *via* their O atoms. A slightly distorted trigonal-bipyramidal SnC_3_O_2_ coordination geometry arises for the metal, with the O atoms in the axial sites. Weak C—H⋯O hydrogen bonds help to stabilize the packing.

## Related literature

For biological activity of tin complexes with carboxyl­ate ligands, see, for example: Shahzadi *et al.* (2007 ▶). For a related structure, see: Danish *et al.* (2009 ▶). For a review of the structural chemistry of tin(IV) complexes with carboxyl­ate ligands, see: Tiekink (1991 ▶). 
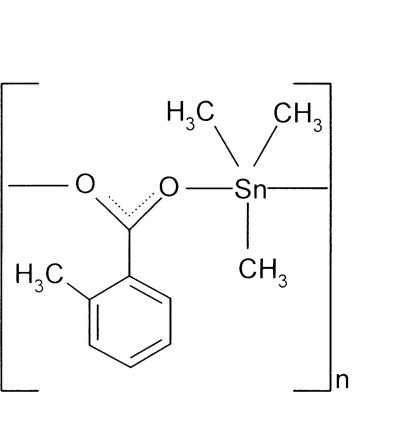


## Experimental

### 

#### Crystal data

[Sn(CH_3_)_3_(C_8_H_7_O_2_)]
*M* *_r_* = 298.93
Monoclinic, 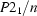

*a* = 10.618 (2) Å
*b* = 10.046 (2) Å
*c* = 12.833 (3) Å
β = 112.39 (3)°
*V* = 1265.7 (4) Å^3^
*Z* = 4
Mo *K*α radiation
μ = 2.00 mm^−1^
*T* = 293 K
0.42 × 0.12 × 0.09 mm

#### Data collection

Kuma KM-4 four circle diffractometer
Absorption correction: analytical (*CrysAlis RED*; Oxford Diffraction, 2008 ▶) *T*_min_ = 0.838, *T*_max_ = 0.892
3389 measured reflections
3226 independent reflections
2090 reflections with *I* > 2σ(*I*)
*R*_int_ = 0.022
3 standard reflections every 200 reflections
intensity decay: 6.4%

#### Refinement

*R*[*F* ^2^ > 2σ(*F* ^2^)] = 0.039
*wR*(*F* ^2^) = 0.118
*S* = 1.04
3226 reflections
131 parameters
H-atom parameters constrained
Δρ_max_ = 1.37 e Å^−3^
Δρ_min_ = −1.69 e Å^−3^

### 

Data collection: *KM-4 Software* (Kuma, 1996 ▶); cell refinement: *KM-4 Software*; data reduction: *DATAPROC* (Kuma, 2001 ▶); program(s) used to solve structure: *SHELXS97* (Sheldrick, 2008 ▶); program(s) used to refine structure: *SHELXL97* (Sheldrick, 2008 ▶); molecular graphics: *SHELXTL* (Sheldrick, 2008 ▶); software used to prepare material for publication: *SHELXL97*.

## Supplementary Material

Crystal structure: contains datablocks I, global. DOI: 10.1107/S1600536809051587/hb5258sup1.cif

Structure factors: contains datablocks I. DOI: 10.1107/S1600536809051587/hb5258Isup2.hkl

Additional supplementary materials:  crystallographic information; 3D view; checkCIF report

## Figures and Tables

**Table 1:** Selected bond lengths (Å)

Sn1—C11	2.108 (6)
Sn1—C12	2.112 (5)
Sn1—C13	2.116 (5)
Sn1—O2^i^	2.200 (3)
Sn1—O1	2.413 (3)

Symmetry code: (i) 
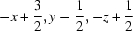
.

**Table 2:** Hydrogen-bond geometry (Å, °)

*D*—H⋯*A*	*D*—H	H⋯*A*	*D*⋯*A*	*D*—H⋯*A*
C13—H13*C*⋯O1^i^	0.96	2.66	3.271 (7)	122
C4—H4⋯O2^ii^	0.93	2.74	3.502 (6)	140

Symmetry codes: (i) 
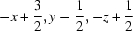
; (ii) 
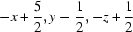
.

**Fig. 1.**
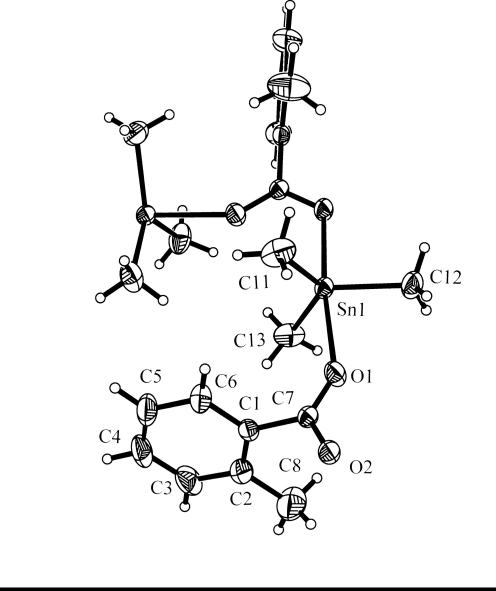
A structural unit of (1) with 50% probability displacement ellipsoids.

**Fig. 2.**
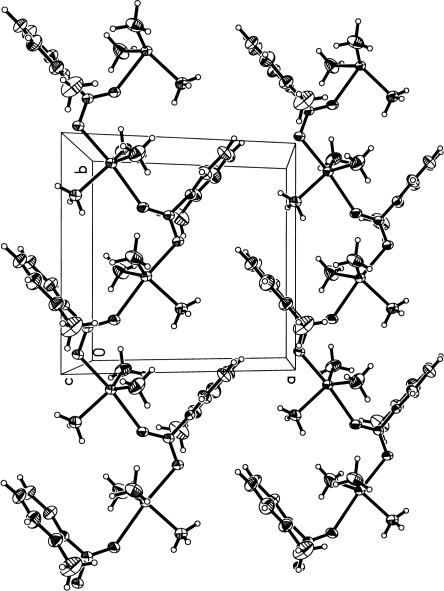
Packing diagram of the structure.

## supplementary crystallographic information

### Comment

The structure of the title compound (I) is built of zigzag molecular chains in which trimethyl-tin units are bridged by toluene-3-carboxylate ligand molecules *via* both their carboxylate O atoms (Fig.1). The toluene ring is planar [r.m.s. 0.0068 (2) Å]. The carboxylic group makes with it a dihedral angle of 77.8 (2)°. A catenated molecular pattern is formed as shown in Fig. 2. Three methyl groups and the tin ion are coplanar [r.m.s. 0.0459 (2) Å]. The metal ion is shifted from the plane by 0.0918 (2) Å. Methyl C atoms form an equatorial plane of a trigonal bipyramid with the bridging carboxylate O atoms at the apices above and below this plane. The chains are kept together by weak hydrogen bonds in which methyl C atoms act as donors and the carboxylate O atoms in the adjacent chains -as acceptors. Weak intra-chain hydrogen bonds are also observed. Geometrical parameters of hydrogen bonds are listed in Table 1.

### Experimental

0.01 mol of sodium *ortho*-toluate was suspended in 25 ml of dry chloroform contained in a round-bottom flask under argon; 0.01 mol of trimethyl-tin chloride was then added with constant stirring. The reacting mixtured was refluxed for 4 h under argon, cooled to room temperature and kept in an ice bath for 1 h. Sodium chloride formed during the reaction was removed by filtration. The filtrate was warmed with activated charcoal for 5 minutes, filtered through silica gel and concentrated to 10 ml. Crude crystals appeared in three days. Then, they were recrystalized from 3:1 chloroform/acetone mixture to yield colourless blocks of (I).

### Refinement

The H atoms attached to toluene-ring C atoms and methyl C atoms were positioned geometrically and refined with a riding model.

### Crystal data

**Table:**

[Sn(CH_3_)_3_(C_8_H_7_O_2_)]	*F*(000) = 592
*M**_r_* = 298.93	*D*_x_ = 1.569 Mg m^−^^3^
Monoclinic, *P*2_1_/*n*	Mo *K*α radiation, λ = 0.71073 Å
Hall symbol: -P 2yn	Cell parameters from 25 reflections
*a* = 10.618 (2) Å	θ = 6–15°
*b* = 10.046 (2) Å	µ = 2.00 mm^−^^1^
*c* = 12.833 (3) Å	*T* = 293 K
β = 112.39 (3)°	Block, colourless
*V* = 1265.7 (4) Å^3^	0.42 × 0.12 × 0.09 mm
*Z* = 4	

### Data collection

**Table:**

Kuma KM-4 four circle diffractometer	2090 reflections with *I* > 2σ(*I*)
Radiation source: fine-focus sealed tube	*R*_int_ = 0.022
graphite	θ_max_ = 30.1°, θ_min_ = 2.1°
profile data from ω/2θ scans	*h* = 0→13
Absorption correction: analytical (*CrysAlis RED*; Oxford Diffraction, 2008)	*k* = −13→0
*T*_min_ = 0.838, *T*_max_ = 0.892	*l* = −17→15
3389 measured reflections	3 standard reflections every 200 reflections
3226 independent reflections	intensity decay: 6.4%

### Refinement

**Table:**

Refinement on *F*^2^	Primary atom site location: structure-invariant direct methods
Least-squares matrix: full	Secondary atom site location: difference Fourier map
*R*[*F*^2^ > 2σ(*F*^2^)] = 0.039	Hydrogen site location: inferred from neighbouring sites
*wR*(*F*^2^) = 0.118	H-atom parameters constrained
*S* = 1.03	*w* = 1/[σ^2^(*F*_o_^2^) + (0.0777*P*)^2^ + 0.0604*P*] where *P* = (*F*_o_^2^ + 2*F*_c_^2^)/3
3226 reflections	(Δ/σ)_max_ = 0.001
131 parameters	Δρ_max_ = 1.37 e Å^−^^3^
0 restraints	Δρ_min_ = −1.69 e Å^−^^3^

### Special details

**Table:**

Geometry. All e.s.d.'s (except the e.s.d. in the dihedral angle between two l.s. planes) are estimated using the full covariance matrix. The cell e.s.d.'s are taken into account individually in the estimation of e.s.d.'s in distances, angles and torsion angles; correlations between e.s.d.'s in cell parameters are only used when they are defined by crystal symmetry. An approximate (isotropic) treatment of cell e.s.d.'s is used for estimating e.s.d.'s involving l.s. planes.
Refinement. Refinement of *F*^2^ against ALL reflections. The weighted *R*-factor *wR* and goodness of fit *S* are based on *F*^2^, conventional *R*-factors *R* are based on *F*, with *F* set to zero for negative *F*^2^. The threshold expression of *F*^2^ > σ(*F*^2^) is used only for calculating *R*-factors(gt) *etc*. and is not relevant to the choice of reflections for refinement. *R*-factors based on *F*^2^ are statistically about twice as large as those based on *F*, and *R*- factors based on ALL data will be even larger.

### Fractional atomic coordinates and isotropic or equivalent isotropic displacement parameters (Å^2^)

**Table:**

	*x*	*y*	*z*	*U*_iso_*/*U*_eq_	
Sn1	0.67213 (3)	0.58902 (3)	0.23186 (2)	0.04378 (12)	
O1	0.8105 (3)	0.7804 (3)	0.2342 (4)	0.0645 (9)	
C1	1.0203 (4)	0.7136 (4)	0.2204 (4)	0.0443 (8)	
C7	0.9255 (4)	0.8130 (4)	0.2391 (3)	0.0429 (8)	
C4	1.1974 (6)	0.5271 (6)	0.1924 (6)	0.0803 (17)	
H4	1.2582	0.4665	0.1826	0.096*	
C6	1.0906 (6)	0.6253 (6)	0.3062 (5)	0.0651 (13)	
H6	1.0792	0.6290	0.3745	0.078*	
C5	1.1780 (6)	0.5310 (7)	0.2904 (6)	0.0803 (17)	
H5	1.2230	0.4707	0.3475	0.096*	
C2	1.0385 (5)	0.7088 (5)	0.1204 (4)	0.0632 (12)	
C3	1.1262 (7)	0.6142 (7)	0.1067 (6)	0.0825 (19)	
H3	1.1374	0.6092	0.0383	0.099*	
C8	0.9607 (9)	0.8037 (9)	0.0242 (6)	0.110 (3)	
H8A	0.8646	0.7885	0.0011	0.165*	
H8B	0.9873	0.7881	−0.0384	0.165*	
H8C	0.9813	0.8940	0.0494	0.165*	
O2	0.9682 (3)	0.9314 (3)	0.2610 (3)	0.0527 (7)	
C12	0.5145 (5)	0.7309 (5)	0.1961 (6)	0.0780 (17)	
H12A	0.5492	0.8098	0.2399	0.117*	
H12B	0.4420	0.6947	0.2147	0.117*	
H12C	0.4804	0.7529	0.1173	0.117*	
C13	0.7115 (6)	0.5114 (6)	0.0940 (4)	0.0725 (15)	
H13A	0.8034	0.5321	0.1031	0.109*	
H13B	0.6495	0.5504	0.0253	0.109*	
H13C	0.6994	0.4166	0.0909	0.109*	
C11	0.7997 (7)	0.5631 (6)	0.4027 (5)	0.0814 (18)	
H11A	0.8469	0.6448	0.4318	0.122*	
H11B	0.8646	0.4940	0.4090	0.122*	
H11C	0.7456	0.5388	0.4450	0.122*	

### Atomic displacement parameters (Å^2^)

**Table:**

	*U*^11^	*U*^22^	*U*^33^	*U*^12^	*U*^13^	*U*^23^
Sn1	0.04404 (18)	0.03127 (16)	0.05657 (19)	0.00215 (11)	0.01979 (13)	−0.00013 (11)
O1	0.0526 (18)	0.0341 (15)	0.118 (3)	−0.0025 (13)	0.0456 (18)	−0.0094 (17)
C1	0.0366 (18)	0.0333 (18)	0.063 (2)	−0.0039 (15)	0.0193 (17)	−0.0053 (16)
C7	0.045 (2)	0.0317 (18)	0.053 (2)	0.0008 (15)	0.0201 (17)	−0.0006 (16)
C4	0.057 (3)	0.063 (4)	0.122 (5)	0.017 (3)	0.036 (3)	−0.016 (3)
C6	0.061 (3)	0.059 (3)	0.072 (3)	0.018 (2)	0.022 (2)	0.006 (2)
C5	0.063 (3)	0.062 (4)	0.108 (5)	0.024 (3)	0.024 (3)	0.009 (3)
C2	0.066 (3)	0.059 (3)	0.073 (3)	0.012 (2)	0.036 (3)	0.006 (2)
C3	0.079 (4)	0.088 (4)	0.100 (5)	0.011 (3)	0.056 (4)	−0.015 (4)
C8	0.146 (7)	0.121 (6)	0.083 (4)	0.050 (5)	0.067 (4)	0.033 (4)
O2	0.0473 (17)	0.0323 (15)	0.082 (2)	−0.0026 (11)	0.0282 (16)	−0.0035 (13)
C12	0.055 (3)	0.043 (2)	0.135 (5)	0.003 (2)	0.034 (3)	0.017 (3)
C13	0.099 (4)	0.063 (3)	0.067 (3)	−0.021 (3)	0.044 (3)	−0.015 (3)
C11	0.084 (4)	0.087 (4)	0.055 (3)	−0.026 (3)	0.006 (3)	0.001 (3)

### Geometric parameters (Å, °)

**Table:**

Sn1—C11	2.108 (6)	C2—C3	1.388 (7)
Sn1—C12	2.112 (5)	C2—C8	1.530 (8)
Sn1—C13	2.116 (5)	C3—H3	0.9300
Sn1—O2^i^	2.200 (3)	C8—H8A	0.9600
Sn1—O1	2.413 (3)	C8—H8B	0.9600
O1—C7	1.243 (5)	C8—H8C	0.9600
C1—C2	1.370 (7)	O2—Sn1^ii^	2.200 (3)
C1—C6	1.389 (7)	C12—H12A	0.9600
C1—C7	1.500 (5)	C12—H12B	0.9600
C7—O2	1.266 (5)	C12—H12C	0.9600
C4—C5	1.351 (10)	C13—H13A	0.9600
C4—C3	1.383 (10)	C13—H13B	0.9600
C4—H4	0.9300	C13—H13C	0.9600
C6—C5	1.394 (8)	C11—H11A	0.9600
C6—H6	0.9300	C11—H11B	0.9600
C5—H5	0.9300	C11—H11C	0.9600
			
C11—Sn1—C12	116.8 (3)	C4—C3—C2	121.5 (6)
C11—Sn1—C13	124.8 (3)	C4—C3—H3	119.3
C12—Sn1—C13	117.3 (3)	C2—C3—H3	119.3
C11—Sn1—O2^i^	92.6 (2)	C2—C8—H8A	109.5
C12—Sn1—O2^i^	90.09 (17)	C2—C8—H8B	109.5
C13—Sn1—O2^i^	97.02 (17)	H8A—C8—H8B	109.5
C11—Sn1—O1	86.5 (2)	C2—C8—H8C	109.5
C12—Sn1—O1	83.88 (17)	H8A—C8—H8C	109.5
C13—Sn1—O1	89.46 (17)	H8B—C8—H8C	109.5
O2^i^—Sn1—O1	172.68 (11)	C7—O2—Sn1^ii^	119.7 (3)
C7—O1—Sn1	142.4 (3)	Sn1—C12—H12A	109.5
C2—C1—C6	119.6 (4)	Sn1—C12—H12B	109.5
C2—C1—C7	121.0 (4)	H12A—C12—H12B	109.5
C6—C1—C7	119.3 (4)	Sn1—C12—H12C	109.5
O1—C7—O2	121.4 (4)	H12A—C12—H12C	109.5
O1—C7—C1	121.5 (3)	H12B—C12—H12C	109.5
O2—C7—C1	117.1 (4)	Sn1—C13—H13A	109.5
C5—C4—C3	119.4 (5)	Sn1—C13—H13B	109.5
C5—C4—H4	120.3	H13A—C13—H13B	109.5
C3—C4—H4	120.3	Sn1—C13—H13C	109.5
C1—C6—C5	120.4 (6)	H13A—C13—H13C	109.5
C1—C6—H6	119.8	H13B—C13—H13C	109.5
C5—C6—H6	119.8	Sn1—C11—H11A	109.5
C4—C5—C6	120.1 (6)	Sn1—C11—H11B	109.5
C4—C5—H5	120.0	H11A—C11—H11B	109.5
C6—C5—H5	120.0	Sn1—C11—H11C	109.5
C1—C2—C3	119.0 (5)	H11A—C11—H11C	109.5
C1—C2—C8	120.6 (5)	H11B—C11—H11C	109.5
C3—C2—C8	120.4 (6)		

Symmetry codes: (i) −*x*+3/2, *y*−1/2, −*z*+1/2; (ii) −*x*+3/2, *y*+1/2, −*z*+1/2.

### Hydrogen-bond geometry (Å, °)

**Table:**

*D*—H···*A*	*D*—H	H···*A*	*D*···*A*	*D*—H···*A*
C13—H13C···O1^i^	0.96	2.66	3.271 (7)	122
C4—H4···O2^iii^	0.93	2.74	3.502 (6)	140

Symmetry codes: (i) −*x*+3/2, *y*−1/2, −*z*+1/2; (iii) −*x*+5/2, *y*−1/2, −*z*+1/2.

## References

[bb1] Danish, M., Tahir, M. N., Ahmad, N., Raza, A. R. & Ibrahim, M. (2009). *Acta Cryst.* E**65**, m609–m610. 10.1107/S1600536809015475 PMC2977640 21583826

[bb2] Kuma (1996). *KM-4 Software* Kuma Diffraction Ltd. Wrocław, Poland.

[bb3] Kuma (2001). *DATAPROC* Kuma Diffraction Ltd. Wrocław, Poland.

[bb4] Oxford Diffraction (2008). *CrysAlis RED* Oxford Diffraction Ltd, Abingdon, Oxfordshire, England

[bb5] Shahzadi, S., Shahid, K. & Ali, S. (2007). *Russ. J. Coord. Chem.* **33**, 403–411.

[bb6] Sheldrick, G. M. (2008). *Acta Cryst.* A**64**, 112–122. 10.1107/S0108767307043930 18156677

[bb7] Tiekink, E. R. T. (1991). *Appl. Organomet. Chem.* **5**, 1–23.

